# Transition of an estuarine benthic meiofauna assemblage 1.7 and 2.8 years after a mining disaster

**DOI:** 10.7717/peerj.14992

**Published:** 2023-03-14

**Authors:** Gabriel Coppo, Fabiano S. Pais, Tiago O. Ferreira, Ken M. Halanych, Kyle Donnelly, Ana Carolina Mazzuco, Angelo F. Bernardino

**Affiliations:** 1 Grupo de Ecologia Bentônica, Department of Oceanography, Universidade Federal do Espírito Santo, Vitória, Espírito Santo, Brazil; 2 Plataforma de Bioinformática, Instituto René Rachou, FIOCRUZ/Minas, Belo Horizonte, Minas Gerais, Brazil; 3 Escola Superior de Agricultura Luiz de Queiroz, Universidade de São Paulo, Piracicaba, São Paulo, Brazil; 4 Center for Marine Science, University of North Carolina at Wilmington, Wilmington, NC, United States of America

**Keywords:** Environmental DNA, Pollution, Environmental Impact, Benthos, Rio Doce, Estuary, Meiofauna

## Abstract

**Background:**

Estuaries are transitional coastal ecosystems that are threatened by multiple sources of human pollution. In 2015, mining tailings from an upstream dam failure caused massive metal contamination that impacted benthic assemblages on the Brazilian Rio Doce estuary.

**Methods:**

In this study, we investigate and compare meiofaunal assemblages with eDNA metabarcoding 1.7 years (2017) and 2.8 years (2018) after the initial contamination by mine tailings in order to evaluate the continued impact of sediment mine tailing contaminants on the structure of benthic assemblages after the disaster.

**Results:**

The community was dominated by Arthropoda and Nematoda 1.7 yr after the impacts (42 and 29% of meiofaunal sequence reads, respectively) but after 2.8 years Arthropoda (64.8% of meiofaunal sequence reads) and Rotifera (11.8%) were the most common taxa. This continued impact on meiofaunal assemblage revealed a lower phylogenetic diversity (7.8-fold) in 2018, despite overall decrease in metal concentration (Al, Ba, Cr, As, Fe, Zn, Mn, Pb, Cd, Co) in sediments. Our data suggests that differences in benthic assemblages and loss of diversity may be influenced by contaminants in sediments of this estuary, and indicate that broad eDNA assessments are greatly useful to understand the full range of biodiversity changes in dynamic estuarine ecosystems.

## Introduction

Estuaries are considered dynamic and transitional coastal ecosystems with a high variability in environmental conditions. Most of them are highly productive habitats and acts as a nursery for a great diversity of organisms. For this reason, estuaries are considered one of the most valuable ecosystems in the world, providing important ecological services (Costanza et al., 1997; McLeod et al., 2011; Pendleton et al., 2012; Janakiraman et al., 2017; Lana & Bernardino, 2018). Estuarine environments are naturally stressed and variable habitats due to their plasticity of physic-chemical processes that vary in short spatio-temporal scales (*e.g.*, changes in salinity and tide) (Mulik, Sukumaran & Srinivas, 2020). Nonetheless, during the last century, the contamination of estuarine ecosystems became a worldwide problem (Irabien et al., 2008) due to acute and chronic impacts generated by contamination and pollution, which change the composition of animal assemblages closely associated with sedimentary matrix (Alves et al., 2013; Alves et al., 2015; Varzim et al., 2019).

Meiobenthos, or meiofauna, are sediment associated organisms between 50 and 500 µm (Higgins & Thiel, 1988; Meyer, 1990). Invertebrates larger than 1,000 µm may be included in meiofauna if they spend part of their life as interstitial organisms (McIntyre, 1969; Hakenkamp & Palmer, 2000). Meiofauna undertake important ecological roles in estuarine ecosystems, through the biomineralization of organic matter and enhancing nutrient regeneration, linking trophic levels of the food web (Coull, 1999; Kennedy & Jacoby, 1999). Their high sensitivity to anthropogenic inputs make them excellent proxies for estuarine pollution (Coull, 1999), and bioindicator for the management of coastal environment (Ward & Jacoby, 1992). However, environmental changes in estuaries, caused by human activities, can strongly impact meiofauna community structure and functioning (Kennedy & Jacoby, 1999; Elliott & Quintino, 2007), often leading to functional and long-term ecological changes (Gomes et al., 2017). Salinity, organic matter content and sediment grain size, for example, are strongly related to the spatial distribution of meiofaunal organisms (Austen & Warwick, 1989; Coull, 1999; Rutledge & Fleeger, 1993; Walters & Bell, 1994; Gomes & Bernardino, 2020).

Due to the difficulty and labor requirements of accurately identifying meiofauna organisms by traditional morphological identification protocols, these organisms are usually neglected in many biodiversity assessments. However, in recent years there have been considerable advances in applying DNA-based methods using metabarcoding techniques to disentangle biodiversity patterns of microorganisms (Baird & Hajibabaei, 2012; Taberlet et al., 2012), including meiofauna (Tang, Li & Yan, 2012; Faria et al., 2018; Fais et al., 2020). Recent studies have successfully assessed, by environmental DNA (eDNA) metabarcoding, metazoan biodiversity in different marine ecosystems, such as estuaries (Bernardino et al., 2019; Clark et al., 2020), continental shelf (Bakker et al., 2019; MacNeil et al., 2022), and coastal sediment (Aylagas et al., 2018; Jeunen et al., 2018). This approach has proven to be useful in assessing the compositional data from samples containing such organisms, while the eDNA metabarcoding has proven to be a powerful tool to overcome the limitation for meiofaunal morphological identification (Valentini, Pompanom & Taberlet, 2009; Medinger et al., 2010; Gielings et al., 2021).

The use of eDNA to measure and monitor marine and estuarine biodiversity is gaining popularity (Creer et al., 2010; Bik et al., 2012; Brannock & Halanych, 2015; Brannock et al., 2016; Mäechler et al., 2019; Ruppert, Kline & Rahman, 2019; Berry et al., 2020; Clark et al., 2020; Naro-Maciel et al., 2022). Recent metabarcoding studies using eDNA extracted from sediment (Avó et al., 2017; Lanzén et al., 2017; Faria et al., 2018; Nascimento et al., 2018; Bernardino et al., 2019; Fais et al., 2020; Castro et al., 2021; Pawlowski et al., 2022) demonstrated its usefulness to assess marine biodiversity. For the most part of biodiversity, eDNA metabarcoding can be more efficient than traditional morphological-based taxonomy, enable the bulk identification of multiple species in an environmental sample by simultaneously amplifying individual DNA barcodes, which can allow the identification of specimens that are small, cryptic or too degraded for morphological identification (Steyaert et al., 2020). In addition, it can be an effective technique for determining the quality and recovery in ecosystems following anthropogenic disasters, such as metal contamination after a rupture on a mining dam (Chariton et al., 2015; Cordier et al., 2017; Di Battista et al., 2020; Martínez et al., 2020; He et al., 2021; Leasi, Sevigny & Hassett, 2021).

In point of fact, in November 2015 a large mine tailing dam ruptured in SE Brazil, releasing nearly 50 million m^3^ of iron ore tailings into the Rio Doce watershed. The mine tailings load was carried over 600 km downstream reaching the Rio Doce estuary and the Atlantic Ocean, where it severely impacted estuarine and coastal ecosystems nearby (Carmo et al., 2017; Queiroz et al., 2018; Bernardino et al., 2019; Magris et al., 2019; Gabriel et al., 2020a). The tailings, mainly composed of iron oxyhydroxides, were associated to different potentially toxic elements including Mn, Cr, Pb, Hg, As, La, and Sc, which were 24 times higher for Mn (and more than 200 times higher for other metals, such as Zn and Cu) than before the incident (Queiroz et al., 2018; Queiroz et al., 2021). The first impacts of the tailings deposition in the estuary included loss of several macrofaunal benthic organisms (Gomes et al., 2017), contamination of aquatic organisms (Gabriel et al., 2020a; Queiroz et al., 2021) and changes in sediment bioturbation and biogeochemistry (Barcellos et al., 2021; Queiroz et al., 2021; Barcellos et al., 2022). The mine tailings impacted the benthic macrofauna diversity, composition and trophic groups (*e.g.,* loss of surface-dwelling taxa), and these impacts were still observed on macrofauna even after almost four years (Gomes et al., 2017; Gabriel et al., 2020b).

eDNA metabarcoding identified effects of this disaster in the meiofaunal assemblages in the Rio Doce estuary in August 2017, 1.7 years after the tailings spill (Bernardino et al., 2019). At the time, high levels of Fe contamination were detected in the estuary sediment, suggesting that meiofaunal assemblages were partially influenced by environmental filtering from toxicity of highly contaminated sediments, since this metal concentrations acted as significant predictors of changes in dominant meiofaunal taxa (*e.g.,* nematodes, copepods, ostracods and flatworms) (Bernardino et al., 2019). The Fe concentrations significantly increased by two times two days after the impact (Gomes et al., 2017), and in August 2017 continued to be 2–20 times higher compared to preserved (Piraquê-Açu-Mirim estuary) or polluted estuaries, such as the Vitória Bay, located in a metropolitan and industrial area approximately 100 km to the south (Hadlich et al., 2018). As the time passes and the contamination impacts in Rio Doce are reduced, it is expected that these biological communities will exhibit some degree of recovery, which should be detected by long-term monitoring and biodiversity assessments.

Given the highly dynamic nature of the estuarine ecosystems, and the prediction that levels of contaminants in sediments will decrease with time (see Gabriel et al., 2021), we re-evaluated he Rio Doce meiofaunal assemblages 2.8 years (2018) after the initial impact. Our aim was to evaluate the continued impacts on meiofaunal assemblages in response to sediment contamination by metals, through biodiversity assessment and multivariate association. We hypothesized that meiofaunal composition and diversity would be affected by metal concentrations in the impacted estuarine sediments, leading to ecological recovery, and that higher phylogenetic diversity would occur with a reduction on the contaminant levels.

## Material & Methods

### Sampling site and sampling procedures

**Figure 1:**
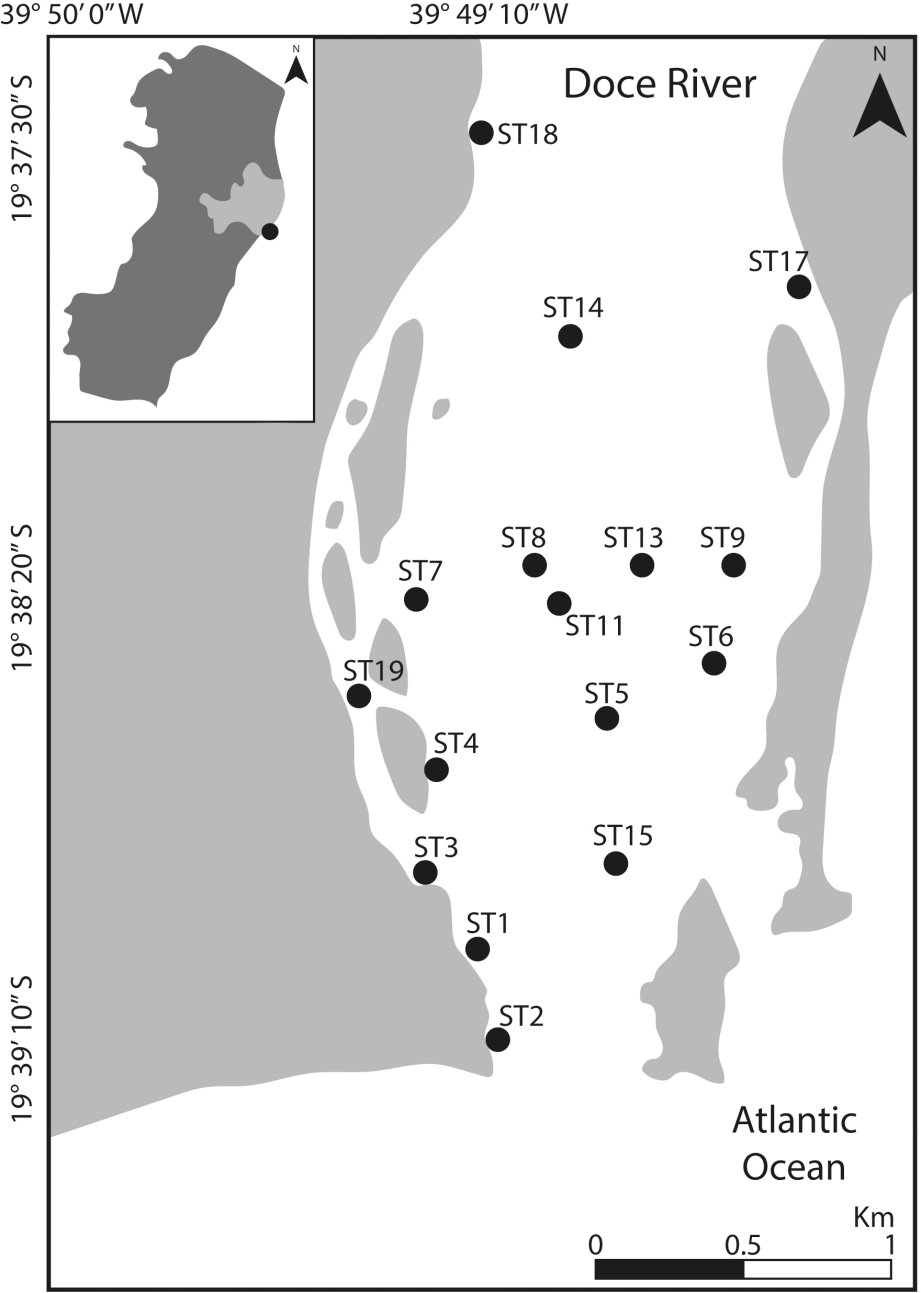
Location of the study area. Map indicating the sampling stations at Rio Doce estuary, on the SE Brazilian coast, in August 2018.

The Rio Doce estuary (19°38′  to 19°45′S, 39°45′ to 39°55′W; Fig. 1) is located in SE Brazil with a tropical climate, and two well-defined seasons, dry winters (April to September) and wet summer (October to March), and a monthly average rainfall of 145 mm (Alvares et al., 2013; Bernardino et al., 2015; Bissoli & Bernardino, 2018). The estuary is characterized by low salinity levels (0.05–8 ppt) and temperatures between 23.1 and 30.5 °C (Gomes et al., 2017; Bernardino et al., 2018; Lana & Bernardino, 2018; Gabriel et al., 2021).

Sampling was carried out in August 2018 at 16 sampling sites distributed throughout the lower portion of the Rio Doce estuary, covering about five km from its mouth (Fig. 1). At each site, we collected two sediment samples (top five cm) using sterile, DNA-free corers and immediately frozen in liquid nitrogen. Additional samples were obtained for determination of grain size, total organic matter and trace metal quantification. All sediment samples were stored in a freezer at −20 °C upon arrival at the laboratory until further analysis. Additionally, water temperature and salinity were measured at each site. Field sampling was approved by SISBIO-IBAMA (sampling license N 24700-1), and data were collected as previously described in Bernardino et al. (2019).

Grain size was determined according to Suguio (1973) by sieving and pipetting, and we quantified total organic matter (TOM) gravimetrically by the weight loss after combustion (500 °C for 3 h). Metal concentration in sediment samples was evaluated from two independent replicate samples. For the total trace metal contents, approximately 1 g of the freeze-dried samples was digested by a tri-acid mixture (nine mL of HNO3 + three mL of HF 1 mol/L + five mL of H_3_BO_3_ 5%; USEPA, 1996) in a microwave oven digestion system. Vessels containing the samples were shaken and heated at 110 °C for 4 h. After that, we diluted samples to 40 mL in deionized water. We determined the concentrations of trace metals (Al, Ba, Cr, As, Fe, Zn, Mn, Pb, Cd, Co) using aliquots of 0.1mL on an ICP-OES spectrometer (iCAP 6200; Thermo Scientific, Waltham, MA, USA; see Queiroz et al., 2018) in triplicate. Standard solutions were prepared from dilution of certified standard solutions and certified reference materials (NIST SRM 2709a) and used for comparison to measured and certified values. Sedimentary and metals concentrations analysis were realized as previously described in Gabriel et al. (2020a).

### DNA extraction and sequencing

Prior to DNA extraction, we elutriated the sediment samples using 45 µm sieves, following the protocol established by Brannock & Halanych (2015), using 950 mL of filtered seawater in a 1L flask, inverting the flask and decanted the liquid over the sieve after the flask was let to sit. After repeating this procedure ten times, sediment retained on the sieve was rinsed to a sterile 50 mL falcon tube, and spun down using an Eppendorf Centrifuge 5430 at room temperature for 3 min at 1,342 × g, and was aliquoted to 20 mL. The sample was mixed using a sterile pipette, and two separate one mL aliquots were removed and stored in separate sterile 1.5 mL tubes, and stored at −20 °C for DNA extraction. All glassware and materials used during the elutriation process were cleaned, sterilized, and autoclaved between samples. After elutriation, we extracted DNA from the sediment samples using the PowerSoil DNA Isolation^®^ kit (Qiagen) following the manufacturer’s instructions. We verified DNA integrity on a 1% agarose gel and purity (260/230 and 260/280 ratios) using a NanoDrop spectrophotometer (Thermo Fisher Scientific Inc., Waltham, MA, USA). We determined DNA concentration using a Qubit^®^ 4 Fluorometer (Life Technologies-Invitrogen, Carlsbad, CA, USA), and samples were sent to ©NGS Genomic Solutions (Piracicaba, SP, Brazil) for metabarcoding sequencing and construction of the amplicon libraries by HiSeq Illumina platform (2 × 250 bp). The V9 hypervariable region of the 18S SSU rRNA gene was amplified using primers Illumina_Euk_1391f forward primer (GTACACACCGCCCGTC) and Illumina_EukBr reverse primer (TGATCCTTCTGCAGGTTCACCTAC) (Medlin et al., 1988; Lane, 1991; Amaral-Zettler et al., 2008; Stoeck et al., 2010).

### Bioinformatic pipelines

We used the 2021.2 QIIME2 software distribution to process and analyze all demultiplexed raw paired-end reads to estimate the observed taxa (Bolyen et al., 2018). Fastq files were first imported as QIIME2 artifacts, and reads were denoised *via* DADA2 (Callahan et al., 2016) with the DADA2 *denoise-paired* plugin, setting the p-trunc parameter to 220 to remove low-quality bases, and the p-trim set to 10 to remove primer sequences.

The taxonomic composition of the amplicon sequence variants (ASV), generated after running the DADA2 plugin, were assigned using the machine learning Python library *scikit-learn* (Pedregosa et al., 2011). The *feature-classifier* plugin was used to generate the classification results by a pre-trained Naïve Bayes classifier trained on Silva 132 database clustered at 99% similarity (Quast et al., 2013), and the taxonomic profiles of each sample were visualized using the *taxa-barplot* plugin. Due to the difference on the number of identified sequences, we normalized datasets from both years to allow analysis and comparison with homogenous sampling depth. We used the 2018 dataset minimum sampling depth (2,282 reads) and resampled each station to the same depth. These filtered/subsampled datasets were used to calculate all diversity metrics.

We reanalyzed and re-identified all sequences from the 2017 assessment realized by Bernardino et al. (2019) following this pipeline to guarantee that both datasets (2017 and 2018 assessments) were treated and analyzed using the same techniques and procedures, and to guarantee we were doing a more accurate comparison. Additionally, we built one phylogenetic tree for each dataset using QIIME2. To generate the trees, we used the *align-to-tree-mafft-fasttree* pipeline from the *q2-phylogeny* plugin. After that, we calculated Faith’s phylogenetic diversity (PD) using the *diversity core-metrics-phylogenetic* pipeline, based on the phylogenetic tree generated before. The PD is obtained summing the branch lengths on a phylogenetic tree, where longer branches correspond to longer evolutionary times and more distinct taxonomic groups (Faith, 1992). Additionally, we plotted rarefaction curves for both assessments. Raw sequence data is deposited in NCBI (SRA: SRR21716030).

### Statistical analysis

Only meiofaunal sequence reads were used for ecological and statistical analysis, and here we considered meiofaunal metazoans the five phyla that are exclusively meiofauna (Gnathostomulida, Kinorhyncha, Loricifera, Gastrotricha, and Tardigrada) and other metazoans that can be representative of meiofauna during any stage of life and play important role in the sediment (temporary meiofauna) (Higgins & Thiel, 1988; Giere, 2009). Normality of all environmental data were tested by Shapiro–Wilk test, and when necessary, data were log-transformed (log10 or log10(x + 1)). Differences in environmental variables, phylogenetic diversity, and the relative abundance of taxa between 2017 and 2018 assessments were assessed by Student’s *t*-test (Student, 1908; Mann & Whitney, 1947). The differences on abundance between phyla were analyzed by a One-Way Analysis of Variance (ANOVA), followed by the Tukey *post-hoc* test for multiple comparisons (Tukey, 1949; Underwood, 1997). A Similarity Percentage Routine (SIMPER) was applied to analyze the contribution of each taxonomic group to the assemblage composition dissimilarity between the two datasets (Clarke, 1993). Linear regressions were performed to evaluate the relation between metals concentrations and phylogenetic diversity and, phyla relative abundances. A non-metrical multidimensional scaling (nMDS; Oksanen et al., 2022) plot was performed with the meiofaunal assemblage composition in August 2017 and August 2018. A canonical analysis of principal coordinates (CAP; Anderson & Willis, 2003) ordination plot was made with the set of environmental variables that better explain the meiofaunal assemblage. Significant differences were defined when *p* < 0.05. All graphical and analytical processes were performed in R environment (R Core Team, 2022).

## Results

### Environmental conditions

In the 2018 assessment, the salinity in the estuary at the time of sampling was 0.14 ± 0.04, and the temperature ranged from 23.7 °C to 26.3 °C. Sediment grain size of sampled stations indicated a predominance of sand particles (minimum = 48.8% and maximum = 94.1%), and the total organic matter (TOM) varied between 1.5 and 11.8% (Table 1; Table S1). We found a significant decrease in concentration of all measured sediment trace metal compared to the assessment made in 2017 (*p* < 0.05; Table 1; Table S1), except for arsenic which increased (*p* = 0.536; Table 1). We measured an average sediment Fe concentration of 16,566 mg/kg. Associated metals, including As, Cr, and Cd still have showed concentrations above the limits allowed by the current legislation (5.9 mg/kg, 37.3 mg/kg, and 0.6 mg/kg, respectively).

**Table 1:** Environmental data from sedimentary samples. Sediment grain size, total organic matter (TOM), and metal concentrations (mg/Kg), as median, minimum and maximum, obtained from sampled station in Rio Doce estuary in August, 2017 and August, 2018. Significant differences (*p* < 0.05) are presented in bold.

Variables	Year				
	2017^*^		2018		*p*
	Median	Min - Max	Median	Min–Max	
%Sand	87.8	11.8–96.2	85.5	48.8–94.1	0.532
TOM	3.20	1.50–16.8	4.00	1.50–11.8	0.646
Al	32,495	10,066–65,386	19,467	10,754–27,590	**<0.001**
As	2.84	<LQ–53.1	4.29	0.15–12.6	0.536
Ba	238.7	33.3–688.4	68.3	26.1–177.3	**<0.001**
Cd	3.25	0.57–7.53	1.76	0.72–2.67	**<0.001**
Co	9.41	3.81–20.9	7.18	4.78–9.69	**0.004**
Cr	47.1	17.7–79.6	25.1	10.25–45.3	**<0.001**
Cu	8.83	2.31–16.1	4.05	0.64–6.65	**<0.001**
Fe	35,538.3	13,204.4–57,923.3	15,990.5	8,981.7–26,862.1	**<0.001**
Mn	551.8	148.4–1094.9	345.3	163.5–539.2	**<0.001**
Ni	14.5	7.17–28.6	10.1	6.27–15.0	**<0.001**
Pb	101.9	4.92–182.2	6.52	3.68–10.9	**<0.001**
Zn	35.4	15.3–85.9	27.4	14.6–46.1	**0.009**

**Notes.**

* Data from August, 2017 were obtained from Bernardino et al. (2019).

### Assemblage structure and phylogenetic diversity

**Figure 2:**
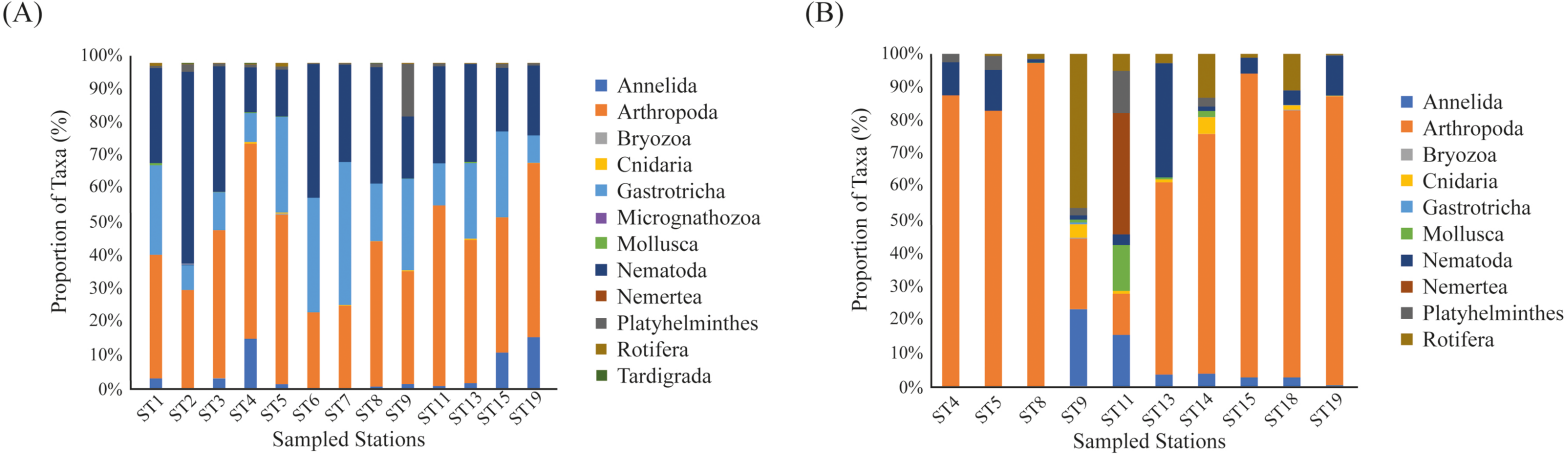
Frequency of identified taxa. Barplots showing (A) the proportion of identified Phylum at the Rio Doce estuary in 2017 assessment, and (B) in the 2018 assessment.

We reanalyzed the data from the 2017 assessment and found a significantly higher number of meiofaunal sequence reads when compared to the 2018 assessment (2017 = 3,090,870 sequence reads; 2018 = 120,627 meiofaunal sequence reads; *t* = 11.147; *p* < 0.001; Table S3). In the 2017 dataset we identified 12 phyla, which is similar to the 10 phyla identified in the 2018 assessment, with the addition of Micrognathozoa, and Tardigrada. The most frequent phyla in the 2017 assemblages were Arthropoda (41.8%) and Nematoda (29.2%) (Fig. 2A).

**Figure 3:**
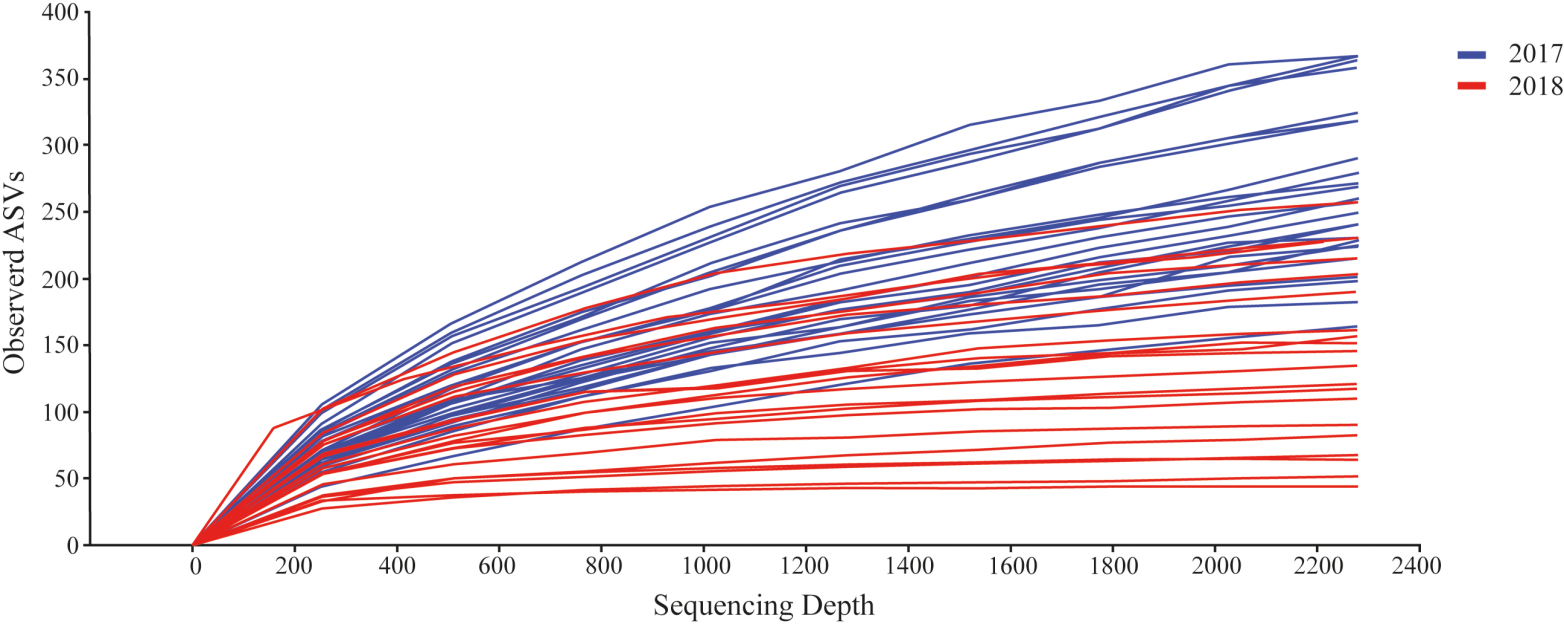
Rarefaction curves from the datasets from 2017 (blue) and 2018 (red).

We detected a total of 162,330 sequences from the eDNA metabarcoding of Rio Doce estuarine sediments in 2018 (Table S2). After filtering the dataset to remove sequences that were not meiofaunal animals (*e.g.*, bacteria, fungi, algae, protists), we obtained 120,627 sequence reads from ten phyla, most of them identified as Arthropoda (64.8% of sequence reads; Table 2) and Rotifera (11.8%; Table 2). The frequencies were significantly different between phyla (*df* = 9; *F* = 12.715; *p* < 0.001; Fig. 2B; Table S3). The rarefaction curves suggest that the number of meiofaunal taxonomic groups was on overall higher in 2017 when compared to 2018 (Fig. 3).

**Table 2:** Frequency of meiofaunal sequences identified in the 2017 and 2018 assessments. Meiofauna assemblage composition and relative frequency of sequences of each amplicon sequence variants (ASVs) identified at Rio Doce estuary in 2017 and 2018 assessments.

**Phylum**	**Class**	**Order**	**2017 Assessment**	**2018 Assessment**
Miscellaneous Annelids			–	5.79%
	Clitellata	Haplotaxida	0.21%	3.09%
		Rhynchobdellida	0.12%	–
	Polychaeta	Echiuroinea	0.00%	–
		Eunicida	1.97%	–
		Spionida	2.73%	0.13%
Miscellaneous Arthropods			0.20%	19.52%
	Arachnida	Acari	0.34%	0.09%
	Branchiopoda		–	0.25%
		Diplostraca	–	<0.01%
	Malacostraca	Eucarida	<0.01%	0.02%
	Maxillopoda		0.02%	7.07%
		Calanoida	0.01%	–
		Hexanauplia(Copepoda)	0.06%	–
		Cyclopoida	–	0.57%
		Harpacticoida	0.10%	0.13%
	Ostracoda	Halocyprida	–	0.02%
		Podocopida	33.68%	25.39%
Bryozoa	Gymnolaemata		0.03%	–
	Phylactolaemata	Plumatellida	–	0.08%
Cnidaria	Anthozoa	Actiniaria	<0.01%	–
		Zoantharia	0.01%	–
	Hydrozoa		0.01%	0.01%
		Anthoathecata	0.06%	0.02%
		Limnomedusae	0.01%	2.45%
	Myxozoa	Bivalvulida	0.01%	0.08%
Gastrotricha		Chaetonotida	23.11%	0.12%
Micrognathozoa			<0.01%	–
Miscellaneous Molluscs			–	<0.01%
	Bivalvia		–	0.08%
		Myoida	0.04%	–
		Nuculoida	<0.01%	–
		Veneroida	0.01%	2.82%
	Gastropoda	Caenogastropoda	0.06%	–
		Heterobranchia	<0.01%	–
Nematoda	Chromadorea		0.08%	0.09%
		Aerolaimida	0.03%	–
		Chromadorida	0.02%	–
		Desmodorida	6.43%	0.23%
		Monhysterida	16.73%	4.52%
		Rhabditida	<0.01%	–
		Tylenchida	<0.01%	0.02%
	Enoplea	Dorylaimia	0.22%	1.39%
		Enoplida	6.72%	0.16%
		Triplonchida	0.38%	0.56%
Nemertea	Anopla	Heteronemertea	<0.01%	5.84%
	Enopla	Monostilifera	–	0.09%
Miscellaneous Platyhelminthes			0.02%	0.62%
	Catenulida		0.01%	0.31%
	Monogenea	Monopisthocotylea	0.07%	0.10%
	Rhabditophora	Macrostomida	0.01%	1.97%
		Proseriata	0.91%	–
		Rhabdocoela	5.13%	0.47%
		Seriata	0.13%	0.03%
	Trematoda		–	0.18%
		Echinostomida	–	0.01%
Rotifera	Bdelloidea		<0.01%	14.90%
		Adinetida	–	0.05%
		Philodinida	–	0.09%
	Monogononta		<0.01%	0.60%
		Flosculariacea	0.01%	–
		Ploimida	0.23%	0.04%
Tardigrada	Eutardigrada	Parachela	0.03%	–

Further, we observed a significant decrease in phylogenetic diversity (PD) from 2017 to 2018. Meiofaunal assemblages in 2017 had a mean PD of 166.6 ± 35.1, when compared to the meiofaunal PD in 2018 of 21.3 ± 7.2; a significant decrease in PD of 7.8 times in 2018 (*t* = 23.320, *df* = 44, *p* < 0.001). In addition, we observed the same pattern for Shannon diversity, with significant higher diversity in 2017 (2017 dataset = 5.46 ± 0.48, and 2018 dataset = 4.75 ±  0.79; *df* = 21; *t* = 2.639; *p* = 0.015).

**Figure 4:**
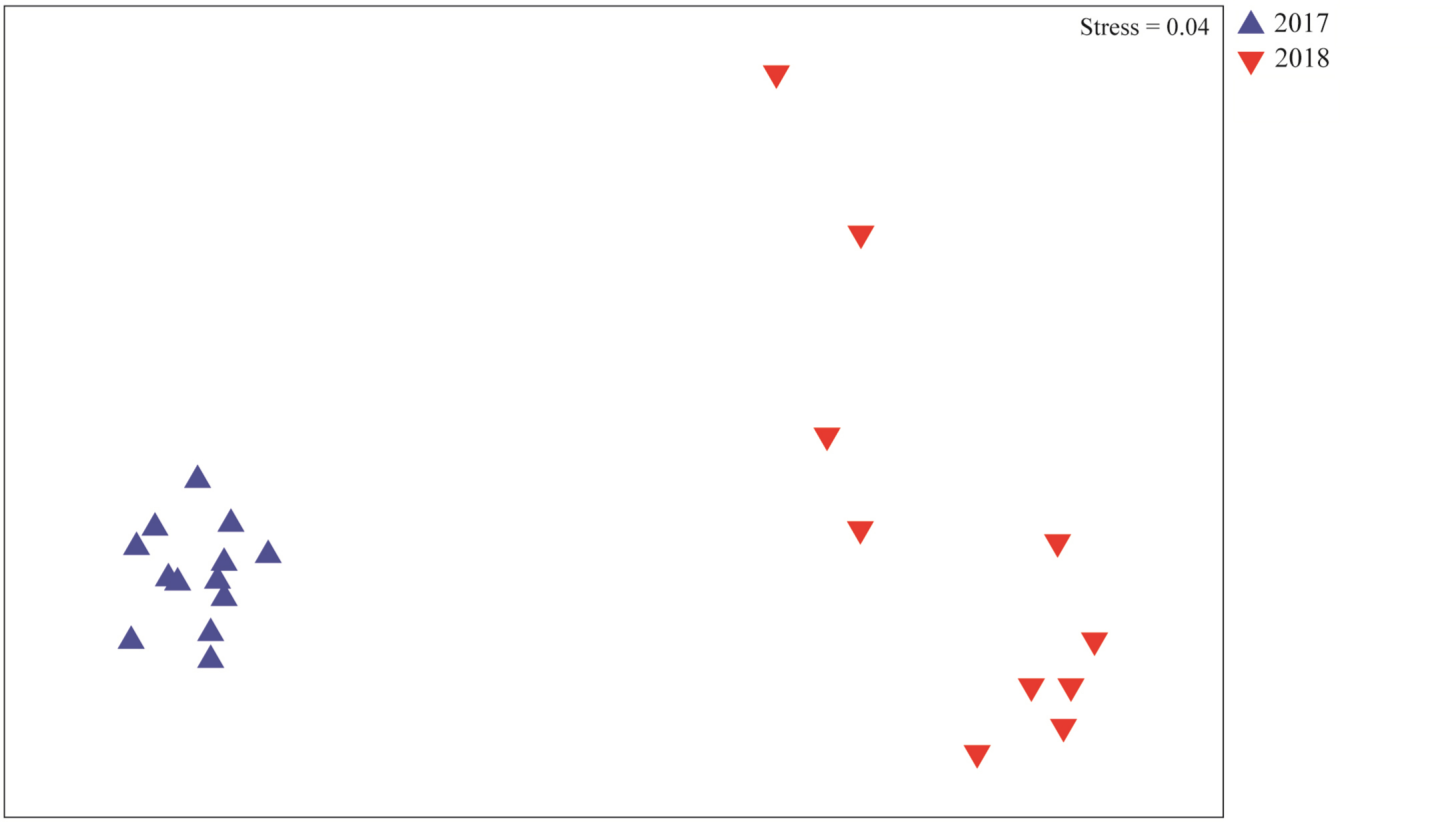
Meiofaunal assemblages composition. Non-metrical multidimensional scaling (nMDS) plot based on meiofaunal assemblage composition in August 2017 (blue triangles) and August 2018 (red triangles).

Multivariate analysis revealed significant differences on the composition of meiofauna assemblages in the Rio Doce estuary between years (Fig. 4). The phyla that most contributed to this difference are Nematoda (24%), Gastrotricha (23.3%), and Arthropoda (18.9%); which contributed to 49.25% of the dissimilarity between the 2017 and 2018 assemblages (Table 3).

**Table 3:** SIMPER results. Results from similarity percentage analysis (SIMPER) indicating each Phylum contribution to the similarity between 2017 and 2018 assessment in the Rio Doce estuary.

**Phyla**	**Av. Dissim.**	**Contrib. (%)**	**Cumulative (%)**
Nematoda	11.81	24	24
Gastrotricha	11.48	23.3	47.35
Arthropoda	9.32	18.93	66.27
Rotifera	7.71	15.65	81.92
Nemertea	2.96	6.01	87.93
Annelida	1.99	4.04	91.97
Mollusca	1.39	2.82	94.79
Platyhelminthes	1.29	2.63	97.42
Cnidaria	1.22	2.49	99.91
Bryozoa	0.02	0.05	99.96
Tardigrada	0.01	0.03	99.99
Micrognathozoa	0.00	0.01	100.00

**Notes.**

Av. Dissim. Average Dissimilarity Contrib. Contribution

### Association with metals and sediments

**Figure 5:**
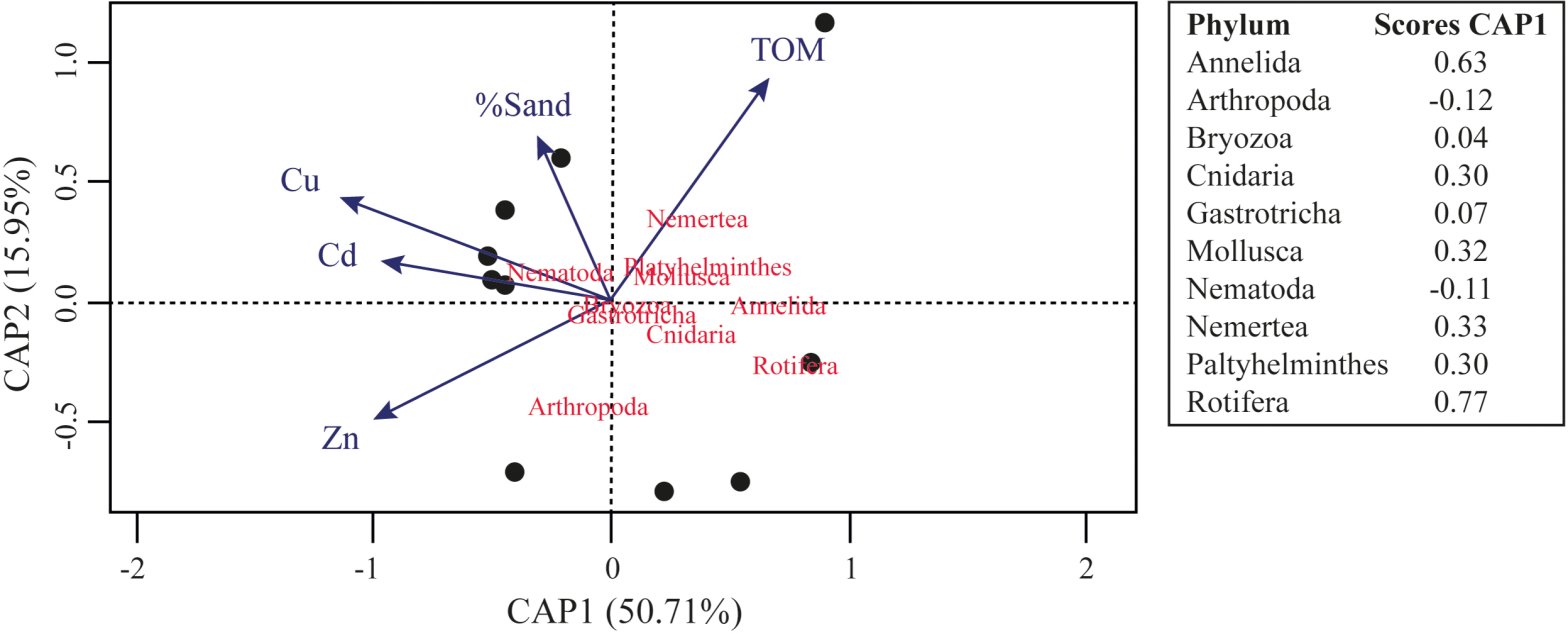
Canonical analysis of principal coordinates (CAP) ordination of samples according to multivariate distribution of the meiofaunal metazoans identified in the Rio Doce estuary in 2018.

The results of assemblages’ composition in 2018 have a negative relation between the Al concentration and the relative abundance of Mollusca ASVs (*F* = 4.964; *R*^2^ = 0.209; *p* = 0.043) and Platyhelminthes ASVs (*F* = 4.408; *R*^2^ = 0.185; *p* = 0.050). Furthermore, we observed significant negative relation between the Zn concentration (*F* = 14.31; *R*^2^ = 0.412; *p* = 0.001), Ni concentration (*F* = 9.877; *R*^2^ = 0.318; *p* = 0.006), Pb concentration (*F* = 7.302; *R*^2^ = 0.249; *p* = 0.015), Co concentration (*F* = 13.11; *R*^2^ = 0.389; *p* = 0.002) and phylogenetic diversity. Even other negative relationships were observed between phyla ASVs and metals concentrations, or between Faith’s Phylogenetic Diversity and metals concentrations, they were not significative. The CAP analysis demonstrated that TOM, %Sand, Zn, Cu and Cd is the best set of variables to explain the distribution of meiofaunal assemblage in 2018, and this model significatively explain 66.66% of the distribution of the identified meiofaunal metazoans (Fig. 5; *F* = 2.378; *p* = 0.044).

**Figure 6:**
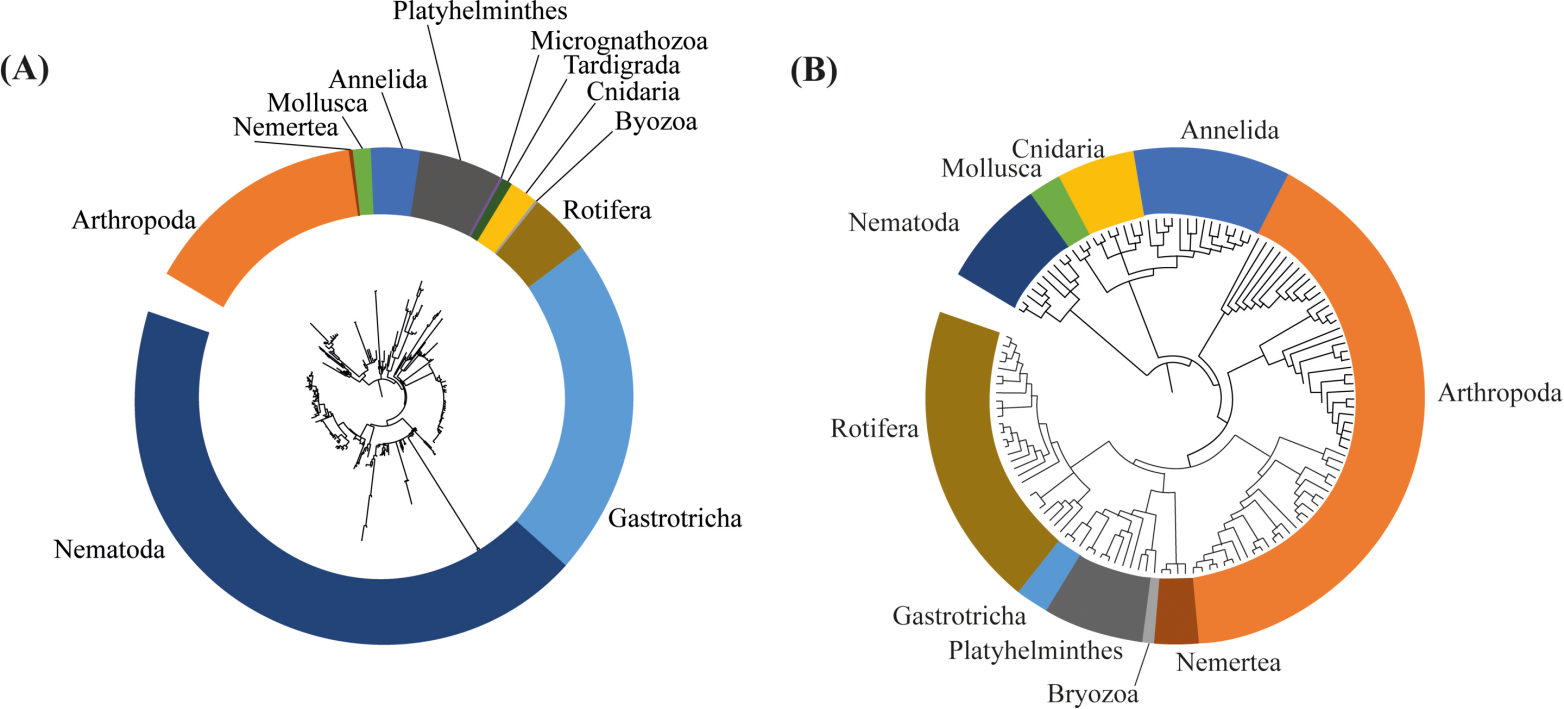
Phylogenetic trees based on the amplicon sequence variants (ASVs) identified from (A) 2017, and (B) 2018 assessments in the Rio Doce estuary.

Differences in the composition of assemblages, and in the phylogenetic diversity between 2017 and 2018 can also be observed on the respective phylogenetic trees, built based on the ASVs identified in the dataset of each sampling event. We can observe a more complex, diverse and with longer branches in the tree based on the 2017 assessment (Fig. 6A). In the phylogenetic tree from 2017 the branches are longer and more divided in different nodes, representing more diversity, especially in Nematoda, Gastrotricha and Platyhelminthes. Additionally, in 2018 the meiofaunal assemblage changed, since the branches are shorter and less divided in different nodes. Is notable how Arthropoda and Rotifera become more representative phyla for the assemblage composition (Fig. 6B).

## Discussion

eDNA metabarcoding of the Rio Doce estuary revealed a lower meiofaunal phylogenetic diversity 2.8 years after the mine tailing disaster, which is contrary to our initial hypothesis of a temporal increase of meiofaunal diversity along an expected decrease in sediment contamination. The temporal comparison of meiofauna assemblages showed significant changes in the composition and diversity (Figs. 2; 6) of meiofaunal organisms, which are markedly associated with the metal contamination in the sediments. Therefore, our results support that the meiofaunal assemblage in the Rio Doce estuary has changed substantially between 2017 and 2018, but with observed reductions in phylogenetic diversity, number of sequences, and changes in the relative abundance of each taxon.

Sediment metal concentrations decreased since the initial impacts were observed in the Rio Doce estuary, but concentrations are still well above pre-impact levels (Gomes et al., 2017; Gabriel et al., 2021). Estuaries are commonly considered ecosystems with low diversity, due to the highly dynamic hydrological conditions (Gray, Wu & Or, 2002; Anila Kumary, 2008; Alves et al., 2013; Janakiraman et al., 2017; Hadlich et al., 2018). Nematodes and Arthropoda are common taxa in estuarine sediments (Coull, 1999; Dalto & Albuquerque, 2000), and in the Rio Doce they represented over 70% of the taxa sampled (Table 2; Fig. 2). These taxa were key to differences observed between 2017 and 2018. In 2017, Nematoda was dominant in the same sampled stations representing 29.2% of sequences of meiofauna (Bernardino et al., 2019).

Copepods are known as a pollution sensitive taxon (Won et al., 2018), but nematodes are highly tolerant to pollution, and some species detoxify absorbed or ingested metals by using metal-binding proteins (Montserrat et al., 2003; Ferraro et al., 2006). Millward & Grant (1995) applied toxicity tests on a nematode community from a severely contaminated estuary, and evidenced that nematodes are resistant to Cu. Thus, the higher dominance of nematodes in 2017 may be related to the higher levels of metals (Bernardino et al., 2019); and their decreased abundance in 2018 suggests a temporal succession of dominance; possibly related to a gradual decrease in pollution observed in the estuary (see Gabriel et al., 2021). This reduction on the relative abundance of nematodes (from 29.2% to 5.2% of total meiofaunal sequences), which are a potential indicator of contaminated sediments, may indicate an assemblage response to the reduction in the metal concentrations in the sediment, where other less tolerant taxa can compete with taxa that are more tolerant to toxicity.

The significant changes observed in meiofaunal assemblages supports the marked temporal changes in environmental conditions of the estuarine sediments. We additionally observed a stronger degree of dissimilarity in assemblages in 2018, which support high bottom heterogeneity and some recovery. The higher heterogeneity in sediment composition can be a source of species nestedness (or loss) in estuarine sediments (Menegotto, Dambros & Netto, 2019), which could explain lower taxonomic diversity and higher dominance of Arthropods in 2018.

The distribution of metals (*e.g.*, Cd, Cu and Zn) may help explain the distribution pattern of meiofaunal metazoans in 2018. McLeese, Sprague & Ray (1987) indicated Cd as not toxic at typical environmental concentrations. Some other studies on meiofauna suggest that Cd does not affect species compositions (Austen & McEvoy, 1997; Austen & Somerfield, 1997). Trannum et al. (2004) did not observe negative effects from high concentrations of Cd on the recolonization of different benthic taxa. On the other hand, Wakkaf et al. (2020) observed Cd toxicity to meiobenthic nematodes. Copper, a common contaminant in bays and estuaries (Hadlich et al., 2018), and considered to be most toxic metal to many marine species (NAS, 1977), showed negative correlations with benthic recolonization rates in experiments realized by Olsgard (1999) and Trannum et al. (2004). Although Zn is not considered toxic to marine organisms (Bryan & Langston, 1999), Gyedu-Abadio (2011) found influences of this metal on the structure of nematodes in two estuaries in South Africa.

Metal concentrations had a significant effect on meiofaunal assemblages after 2.8 years, in addition to sedimentary organic content and grain size. Organic matter contents in the sediment plays a key role and is a nutrient source that determine benthic organisms’ distribution (Paarsons, Takahashi & Hargrave, 1984; Neto, Bernardino & Netto, 2021). The distribution of some meiofaunal organisms may be influenced by grain size, like crustaceans that are usually more abundant in coarse sediments (Tietjen, 1969; Hicks & Coull, 1983). Grain size determines structural and spatial conditions from the habitat, and indirectly influences the physical and chemical parameters of it (Giere, 2009). In fact, different studies suggest that abiotic factors, such as grain size and organic matter content, contribute to the patchy distribution of meiofaunal assemblages in a similar pattern observed in the present study (Nascimento, Karlson & Elmgren, 2008; Alves et al., 2009; Faria et al., 2018; Fais et al., 2020).

We expected to detect a meiofaunal successional process towards assemblages with higher richness and diversity, when compared to the 2017 assessment, which would suggest a recovery process from chronic impacts of metal contamination. Our study then supports that other factors can influence the rate at which biotic assemblages recover from environmental disasters. Our results suggest that the Rio Doce estuary was not yet on a recovery path after nearly 3 years from the initial impacts, as ecosystems are not considered recovered until a secondary succession returns the ecosystem to the pre-existing condition (Borja, Dauer & Elliott, 2010). In this sense, we would need continued long-term assessments to determine its a recovery trajectory (Latimer et al., 2003). The recovery of benthic communities can vary greatly from weeks (Danovaro, Fabiano & Vincx, 1995) to 10–25 years (Jones & Schmitz, 2009; Borja, Dauer & Elliott, 2010; Aderhold et al., 2018), and some ecosystems may never be technically recovered and end up irreversibly in an alternative state (Borja, Dauer & Elliott, 2010). A similar result was observed by Fleeger et al. (2019) that did not observe a full recover on meiofaunal assemblages 6.5 years after an oil spill contamination. Our results corroborate those found by Gambi et al. (2020) that clearly detected the effect of long-term tailing discharge on benthic diversity after several decades from the end of the mining. In our case, it is difficult or even impossible to determine the state of recovery the Rio Doce estuary since there are no baseline data or long-term studies of meiofaunal assemblages in this estuary.

The meiofaunal phylogenetic diversity from the Rio Doce estuary suggests losses of diversity in assemblage composition from 1.7 to 2.8 years after initial impacts. This may be a result or a response to the chronic effects of the metal concentrations following the disaster since, despite a significant decrease on metal concentrations, the contamination remains above reference values (Gabriel et al., 2021). These observed differences in meiofauna assemblages may indicate changes in other biological components, and consequently in the whole estuarine ecosystem. The loss of some meiofauna phyla and the decrease in phylogenetic biodiversity corroborates to this hypothesis.

## Conclusion

In conclusion, we observed substantial differences on meiofaunal assemblage composition and diversity in the Rio Doce estuary from 1.7 yrs to 2.8 yrs after a mine tailing disaster. Although sediment metal concentrations decreased in time, we observed fewer identified sequences and phylogenetic diversity. Our results suggest that meiofaunal diversity are now influenced by total organic matter content and grain size, but the continuous contamination by trace metals including Cd, Cu and Zn seems to still influence assemblage diversity. On the other hand, the reduction on Nematoda relative abundance—a tolerant taxa to toxicity—may indicate a recovery of meiofaunal assemblages *via* competition with less tolerant taxa. Additionally, we reinforce that the use of eDNA assessments is very useful and cost-effective to understand the dynamic of estuarine ecosystems and temporal changes on biodiversity. The continued sampling and monitoring on the Rio Doce estuary would be of great importance to understand how this meiofaunal assemblage will respond during the successional process over time.

##  Supplemental Information

10.7717/peerj.14992/supp-1 Supplemental Information 1 Meiofaunal sequences from Rio Doce estuary Number of sequence variant reads from meiofaunal taxa from the Rio Doce estuary in August 2018, distributed across sampled sites.

10.7717/peerj.14992/supp-2 Supplemental Information 2 Raw data of sedimentary variables Raw data (per sampled station) of sedimentary variables obtained from sediment samples from the Rio Doce estuary 2017 and 2018 assessments.

10.7717/peerj.14992/supp-3 Supplemental Information 3 Student’s *t*-test results Results of Student’s *t*-test comparing environmental variables between 2017 and 2018 sampling in the Rio Doce estuary.

10.7717/peerj.14992/supp-4 Supplemental Information 4 Sampling stations and number of sequences Latitude, Longitude, salinity, and number of sequences (after filtering) identified as meiofaunal metazoans in each sampled station in the 2018 assessment in the Rio Doce estuary.

10.7717/peerj.14992/supp-5 Supplemental Information 5 ANOVA results for meiofaunal sequences Results of ANOVA comparing the frequencies of meiofaunal representative sequences obtained in the 2018 assessment in the Rio Doce estuary.

## Additional Information and Declarations

### Field Study Permissions

The following information was supplied relating to field study approvals (i.e., approving body and any reference numbers): Field sampling was approved by the SISBIO-IBAMA (sampling license N 24700-1).

### DNA Deposition

The following information was supplied regarding the deposition of DNA sequences: The sequences are available at NCBI SRA: SRR21716030, SAMN31026701. The RioDoce18ST data is also available at NCBI: SRR21716030. The data is also available at FigShare: Coppo, Gabriel (2022): riodoce18. figshare. Dataset. https://doi.org/10.6084/m9.figshare.21133567.v1.
https://trace.ncbi.nlm.nih.gov/Traces/?view=run_browser&page_size=10&acc=SRR21716030&display=download.

### Data Availability

The following information was supplied regarding data availability: The raw data is available in the Supplementary Files and Table 1.

## References

[ref-1] Aderhold DGR, Lindenberg MR, Holderied K, Pegau WS (2018). Spatial and temporal ecological variability in the northern Gulf of Alaska: what have we learned since the Exxon Valdez oil spill?. Deep Sea Research Part II.

[ref-2] Alvares CA, Stape JL, Sentelhas PC, Gonçalves JLM, Sparovek G (2013). Koppen’s climate classification map for Brazil. Metereologische Zeitschrift.

[ref-3] Alves AS, Adão H, Ferrero TJ, Marques JC, Costa MJ, Patrício J (2013). Benthic meiofauna as indicator of ecological changes in estuarine ecosystems: the use of nematodes in ecological quality assessment. Ecological Indicators.

[ref-4] Alves AS, Adão H, Patrício J, Neto JM, Costa MJ, Marques JC (2009). Spatial distribution of subtidal meiobenthos along estuarine gradients in two southern European estuaries (Portugal). Acta Oecologica.

[ref-5] Alves AS, Caetano A, Costa JL, Costa MJ, Marques JC (2015). Estuarine intertidal meiofauna and nematode communities as indicator of ecosystem’s recovery following mitigation measures. Ecological Indicators.

[ref-6] Amaral-Zettler L, Peplies J, Ramette A, Fuchs B, Ludwig W, Glöckner FO (2008). Proceedings of the international workshop on Ribosomal RNA technology, April (2008) 7–9, Bremen, Germany. Systematic and Applied Microbiology.

[ref-7] Anderson MH, Willis TJ (2003). Canonical analysis of principal coordinates: a useful method of constrained ordination for ecology. Ecology.

[ref-8] Anila Kumary KS (2008). Diversity of meiobenthic nematodes in the Poonthura estuary (southwest coast of India). Journal of the Marine Biological Association of India.

[ref-9] Austen MC, McEvoy AJ (1997). The use of offshore meiobenthic communities in laboratory microcosm experiments: response to heavy metal contamination. Journal of Experimental Marine Biology and Ecology.

[ref-10] Austen MC, Somerfield PJ (1997). A community level sediment bioassay applied to an estuarine heavy metal gradient. Marine Environmental Research.

[ref-11] Austen MC, Warwick RM (1989). The specificity of meiobenthic community response to different pollutants: results from microcosm experiments. Marine Pollution Bulletin.

[ref-12] Avó AP, Daniell TJ, Neilson R, Oliveira S, Branco J, Adão H (2017). DNA barcoding and morphological identification of benthic nematodes assemblages of estuarine intertidal sediments: advances in molecular tools for biodiversity assessment. Frontiers in Marine Science.

[ref-13] Aylagas E, Borja Á, Muxika I, Rodríduez-Ezpeleta N (2018). Adapting metabarcoding-based benthic biomonitoring into routine marine ecological status assessment networks. Ecological Indicators.

[ref-14] Baird D, Hajibabaei M (2012). Biomonitoring 2.0: a new paradigm in ecosystem assessment made possible by next-generation DNA sequencing. Molecular Ecology.

[ref-15] Bakker J, Wangensteen OS, Baillie C, Buddo D, Chapman DD, Gallagher AJ, Guttridge TL, Hertler H, Mariani S (2019). Biodiversity assessment of tropical shelf eukaryotic communities via pelagic eDNA metabarcoding. Ecology and Evolution.

[ref-16] Barcellos D, Jensen SSK, Bernardino AF, Gabriel FA, Ferreira TO, Quintana CO (2021). Benthic bioturbation: a canary in the mine for the retention and release of metals from estuarine sediments. Marine Pollution Bulletin.

[ref-17] Barcellos D, Queiroz HM, Ferreira AD, Bernardino AF, Nobrega GN, Otero XL, Ferreira TO (2022). Short-term Fe reduction and metal dynamics in estuarine soils impacted bt Fe-rich mine tailings. Applied Geochemistry.

[ref-18] Bernardino AF, Netto SA, Pagliosa PR, Barros F, Christofoletti RA, Rosa-Filho JS, Colling J, Lana PC (2015). Predicting ecological changes on benthic estuarine assemblages through decadal climate trends along Brazilian marine ecoregions. Estuarine, Coastal and Shelf Science.

[ref-19] Bernardino AF, Pais FS, Oliveira LS, Gabriel FA, Ferreira TO, Queiroz HM, Mazzuco AC (2019). Chronic trace metals effects of mine tailings on estuarine assemblages revealed by environmental DNA. PeerJ.

[ref-20] Bernardino AF, Reis A, Pereira Filho ACD, Gomes LEO, Bissoli Jr LB, Barros FCR, Lana PC, Bernardino AF (2018). Benthic estuarine assemblages of the Eastern Marine Brazilian Ecoregion (EME). Brazilian estuaries, Brazilian marine biodiversity.

[ref-21] Berry O, Jarman S, Bissett A, Hope M, Paeper C, Bessey C, Schwartz MK, Hale J, Bunce M (2020). Making environmental DNA (eDNA) biodiversity records globally accessible. Environmental DNA.

[ref-22] Bik HM, Porazinska DL, Creer S, Caporaso JG, Knight R, Thomas WK (2012). Sequencing our way towards understanding global eukaryotic biodiversity. Trends in Ecology & Evolution.

[ref-23] Bissoli LB, Bernardino AF (2018). Benthic macrofaunal structure and secondary production in tropical estuaries on the Eastern Marine Ecoregion of Brazil. PeerJ.

[ref-24] Bolyen E, Rideout JR, Dillon MR, Bokulich NA, Abnet C, Al-Ghalith GA, Alexander H, Alm EJ, Arumugam M, Asnicar F, Bai Y, Bisanz JE, Bittinger K, Brejnrod A, Brislawn CJ, Brown CT, Callahan BJ, Caraballo-Rodríguez AM, Chase J, Cope E, Silva RDa, Dorrestein PC, Douglas GM, Durall DM, Duvallet C, Edwardson CF, Ernst M, Estaki M, Fouquier J, Gauglitz JM, Gibson DL, Gonzalez A, Gorlick K, Guo J, Hillmann B, Holmes S, Holste H, Huttenhower C, Huttley G, Janssen S, Jarmusch AK, Jiang L, Kaehler B, Kang KB, Keefe CR, Keim P, Kelley ST, Knights D, Koester I, Kosciolek T, Kreps J, Langille MGI, Lee J, Ley R, Liu Y-X, Loftfield E, Lozupone C, Maher M, Marotz C, Martin BD, McDonald D, McIver LJ, Melnik AV, Metcalf JL, Morgan SC, Morton J, Naimey AT, Navas-Molina JA, Nothias LF, Orchanian SB, Pearson T, Peoples SL, Petras D, Preuss ML, Pruesse E, Rasmussen LB, Rivers A, Robeson MSII, Rosenthal P, Segata N, Shaffer M, Shiffer A, Sinha R, Song SJ, Spear JR, Swafford AD, Thompson LR, Torres PJ, Trinh P, Tripathi A, Turnbaugh PJ, Ul-Hasan S, Van Der Hooft JJJ, Vargas F, Vázquez-Baeza Y, Vogtmann E, Von Hippel M, Walters W, Wan Y, Wang M, Warren J, Weber KC, Williamson CHD, Willis AD, Xu ZZ, Zaneveld JR, Zhang Y, Zhu Q, Knight R, Caporaso JG (2018). QIIME 2: reproducible, interactive, scalable, and extensible microbiome data science. PeerJ.

[ref-25] Borja Á, Dauer DM, Elliott M (2010). Medium- and long-term recovery of estuarine and coastal ecosystems: patterns, rates, and restoration effectiveness. Estuaries and Coasts.

[ref-26] Brannock PM, Halanych KM (2015). Meiofaunal community analysis by high-throughput sequencing: comparison of extraction, quality filtering, and clustering methods. Marine Genomics.

[ref-27] Brannock PM, Wang L, Ortmann AC, Waits DC, Halanych KM (2016). Genetic assessment of meiobenthic community composition and spatial distribution in coastal sediments along northern Gulf of Mexico. Marine Environmental Research.

[ref-28] Bryan GW, Langston WJ (1999). Bioavailability, Ac-cumulation and effects of heavy metals in sediments with special reference to UK Estuaries: a review. Environmental Pollution.

[ref-29] Callahan BJ, McMurdie PJ, Rosen MJ, Han AW, Johnson AJA, Holmes SP (2016). DADA2: high-resolution sample inference from Illumina amplicon data. Nature Methods.

[ref-30] Carmo FF, Kamino LHY, Junior RT, Campos IC, Carmo FF, Silvino G, Castro KJ, Mauro ML, Rodrigues N, Miranda M, Pinto CEF (2017). Fundão tailings dam failures: the environment tragedy of the largest technological disaster of Brazilian mining in global context. Perspectives in Ecology and Conservation.

[ref-31] Castro LR, Meyer RS, Shapiro B, Shirazi S, Cutler S, Lagos AM, Quiroga SY (2021). Correction to: metabarcoding meiofauna biodiversity assessment in four beaches of Northern Colombia: effects of sampling protocols and primer choice. Hydrobiologia.

[ref-32] Chariton AA, Stephenson S, Morgan MJ, Steven ADL, Colloff MJ, Court LN (2015). Metabarcoding of benthic eukaryote communities predicts the ecological condition of estuaries. Environmental Pollution.

[ref-33] Clark DE, Pilditch CA, Pearman JK, Ellis JI, Zaiko A (2020). Environmental DNA metabarcoding reveals estuarine benthic community response to nutrient enrichment–evidence from an in-situ experiment. Environmental Pollution.

[ref-34] Clarke KR (1993). Non-parametric multivariate analyses of changes in community structure. Australian Journal of Ecology.

[ref-35] Cordier T, Esling P, Lejzerowicz F, Visco J, Ouadahi A, Martins C, Cedhagen T, Pawlowski J (2017). Predicting the ecological quality status of marine environments from eDNA metabarcoding data using supervised machine learning. Environmental Science & Technology.

[ref-36] Costanza R, d’Arge R, de Groot R, Farber S, Grasso M, Hannon B, Limburg K, Naeem S, O’Neill RV, Paruelo J, Raskin RG, Sutton P, van den Belt M (1997). The value of the world’s ecosystem services and natural capital. Nature.

[ref-37] Coull BC (1999). Role of meiofauna in estuarine soft-bottom habitats. Australian Journal of Ecology.

[ref-38] Creer S, Fonseca VG, Porazinska DL, Giblin-Davis RM, Sung W, Power DM, Packer M, Carvalho GR, Blaxter ML, Lambshead PJD, Thomas WK (2010). Ultrasequencing of the meiofaunal biosphere: practice, pitfalls and promises. Molecular Ecology.

[ref-39] Dalto AG, Albuquerque EF (2000). Meiofauna distribution in a tropical estuary of the South-Western Atlantic (Brazil). Vie et Milieu.

[ref-40] Danovaro R, Fabiano M, Vincx M (1995). Meiofauna response to the Agip Abruzzo oil spill in subtidal sediments of the Ligurian Sea. Marine Pollution Bulletin.

[ref-41] Di Battista JD, Reimer JD, Stat M, Masucci GD, Biondi P, De Brauwer M, Wilkinson SP, Chariton AA, Bunce M (2020). Environmental DNA can act as a biodiversity barometer of anthropogenic pressures in coastal ecosystems. Scientific Reports.

[ref-42] Elliott M, Quintino V (2007). The estuarine quality paradox, environmental homeostasis and the difficulty of detecting anthropogenic stress in naturally stressed areas. Marine Pollution Bulletin.

[ref-43] Fais M, Duarte S, Vieira PE, Sousa R, Hajibabaei M, Canchaya CA, Costa FO (2020). Small-scale spatial variation of meiofaunal communities in Lima estuary (NW Portugal) assessed through metabarcoding. Estuarine, Coastal and Shelf Science.

[ref-44] Faith PD (1992). Conservation evaluation and phylogenetic diversity. Biological Conservation.

[ref-45] Faria LC, Di Domenico M, Andrade SCS, Santos MCdos, Fonseca G, Zanol J, Amaral ACZ (2018). The use of metabarcoding for meiofauna ecological patterns assessment. Marine Environmental Research.

[ref-46] Ferraro L, Sprovieri M, Alberico I, Lirer F, Prevedello L, Marsella E (2006). Benthic foraminífera and heavy metals distribution: a case study from the Naples Harbour (Tyrrhenian Sea, Southern Italy). Environmental Pollution.

[ref-47] Fleeger JW, Riggio MR, Mendelssohn IA, Lin Q, Deis DR, Johnson DS, Carman KR, Graham SA, Zengel S, Hou A (2019). What promotes the recovery of Salt Marsh Infauna after oil spills?. Estuaries and Coasts.

[ref-48] Gabriel FA, Ferreira AD, Queiroz HM, Vasconcelos ALS, Ferreira TO, Bernardino AF (2021). Long-term contamination of the Rio Doce estuary as a result of Brazil’s largest environmental disaster. Perspectives in Ecology and Conservation.

[ref-49] Gabriel FA, Hauser-Davis RA, Soares L, Mazzuco ACA, Rocha RCC, Pierre TDS, Saggioro E, Correia FV, Ferreira TO, Bernardino AF (2020a). Contamination and oxidative stress biomarkers in estuarine fish following a mine tailing disaster. PeerJ.

[ref-50] Gabriel FA, Silva AG, Queiroz HM, Ferreira TO, Hauser-Davis RA, Bernardino AF (2020b). Ecological risks of metal and metalloid contamination in the Rio Doce Estuary. Integrated Environmental Assessment and Management.

[ref-51] Gambi C, Dell’Anno A, Corinaldesi C, Martire ML, Musco L, Da Ros Z, Armiento G, Danovaro R (2020). Impact of historical contamination on meiofaunal assemblages: the case study of the Bagnoli-Coroglio Bay (southern Tyrrhenian Sea). Marine Environmental Research.

[ref-52] Gielings R, Fais M, Fontaneto D, Creer S, Costa FO, Renema W, Macher J-N (2021). DNA metabarcoding methods for the study of marine benthic meiofauna: a review. Frontiers in Marine Science.

[ref-53] Giere O (2009). Meiobenthology: the microscopic motile fauna of aquatic sediments.

[ref-54] Gomes LEO, Bernardino AF (2020). Drought effects on tropical estuarine benthic assemblages in Eastern Brazil. Science of the Total Environment.

[ref-55] Gomes LEO, Correa LB, Sá F, Neto RR, Bernardino AF (2017). The impacts of the Samarco mine tailing spill on the Rio Doce estuary, Eastern Brazil. Marine Pollution Bulletin.

[ref-56] Gray JS, Wu RSS, Or YY (2002). Effects of hypoxia and organic enrichment on the coastal marine environment. Marine Ecology Progress Series.

[ref-57] Gyedu-Abadio TK (2011). Pollution status of two river Estuaries in the Eastern Cape, South Africa, based on benthic meiofauna analyses. Journal of Water Resource and Protection.

[ref-58] Hadlich HL, Venturini N, Martins CC, Hatje V, Tineli P, Gomes LEO, Bernardino AF (2018). Multiple biogeochemical indicators of environmental quality in tropical estuaries reveal contrasting conservation opportunities. Ecological Indicators.

[ref-59] Hakenkamp CC, Palmer MA, Jones JB, Mulholland PJ (2000). The ecology of hyporheic meiofauna. Streams and ground waters.

[ref-60] He X, Gilmore SR, Sutherland TF, Hajibabaei M, Miller KM, Westfall KM, Pawlowski J, Abbott CL (2021). Biotic signals associated with benthic impacts of salmon farms from eDNA metabarcoding of sediments. Molecular Ecology.

[ref-61] Hicks GRF, Coull BC (1983). The ecology of marine meiobenthic harpacticoid copepods. Oceanography and Marine Biology: An Annual Review.

[ref-62] Higgins RP, Thiel H (1988). Introduction to the study of Meiofauna.

[ref-63] Irabien MJ, Cearreta A, Leorri E, Gómez J, Viguri J (2008). A 130 year record of pollution in the Suances estuary (southern Bay of Biscay): implications for environmental management. Marine Pollution Bulletin.

[ref-64] Janakiraman A, Naveed MS, Sheriff MAsrar, Altaff K (2017). Ecological restoration assessment of Adyar creek and estuary using meiofaunal communities as ecological indicators for aquatic pollution. Regional Studies in Marine Science.

[ref-65] Jeunen G-J, Knapp M, Spencer HG, Lamare MD, Taylor HR, Stat M, Bunce M, Gemmell NJ (2018). Environmental DNA (eDNA) metabarcoding reveals strong discrimination among diverse marine habitats connected by water movement. Molecular Ecology Resources.

[ref-66] Jones HP, Schmitz OJ (2009). Rapid recovery of damaged ecosystems. PLOS ONE.

[ref-67] Kennedy AD, Jacoby CA (1999). Biological indicators of marine environmental health: meiofauna—a neglected benthic component?. Environmental Monitoring and Assessment.

[ref-68] Lana PC, Bernardino AF (2018). Brazilian estuaries, Brazilian marine biodiversity.

[ref-69] Lane DJ, Stackebrandt E, Goodfellow M (1991). 16S/23S rRNA sequencing. Nucleic acid techniques in bacterial systematics.

[ref-70] Lanzén A, Lekang K, Jonassen I, Thompson EM, Troedsson C (2017). DNA extraction replicates improve diversity and compositional dissimilarity in metabarcoding of eukaryotes in marine sediments. PLOS ONE.

[ref-71] Latimer JS, Boothman WS, Pesch CE, Chmura GL, Pospelova V, Jayaraman S (2003). Environmental stress and recovery: the geochemical record of human disturbance in New Bedford Harbor and Apponagansett Bay, Massachusetts (USA). The Science of Total Environment.

[ref-72] Leasi F, Sevigny JL, Hassett BT (2021). Meiofauna as a valuable bioindicator of climate change in the polar regions. Ecological Indicators.

[ref-73] MacNeil L, Desai DK, Costa M, La Roche J (2022). Combining multi-marker metabarcoding and digital holography to describe eukaryotic plankton across the Newfoundland Shelf. Scientific Reports.

[ref-74] Mäechler E, Little CJ, Wüthrich R, Alther R, Fronhofer EA, Gounand I, Harvey E, Hürlemann S, Walse J-C, Altermatt F (2019). Assessing different components of diversity across a river network using eDNA. Environmental DNA.

[ref-75] Magris RA, Marta-Almeida M, Monteiro JAF, Ban NC (2019). A modelling approach to assess the impact of land mining on marine biodiversity: assessment in coastal catchments experiencing catastrophic events (SW Brazil). Science of the Total Environment.

[ref-76] Mann HB, Whitney DR (1947). On a test of whether one of two random variables is stochastically larger than the other. Annals of Mathematical Statistics.

[ref-77] Martínez A, Eckert EM, Artois T, Careddu G, Casu M, Curini-Galletti M, Gazale V, Gobert S, Ivanenko VN, Jondelius U, Marzano M, Pesole G, Zanello A, Todaro MA, Fontaneto D (2020). Human access impacts biodiversity of microscopic animals in sandy beaches. Communications Biology.

[ref-78] McIntyre AD (1969). Ecology of marine meiobenthos. Biological Reviews.

[ref-79] McLeese DW, Sprague JB, Ray S, Nriagu JO, Sprague JB (1987). Effects of cadmium on marine biota. Cadmium in the Aquatic Environment. Adv. Environ. Sci. Technol.

[ref-80] McLeod E, Chmura GL, Bouillon S, Salm R, Björk M, Duarte CM, Lovelock CE, Schlesinger WH, Silliman BR (2011). A blueprint for blue carbon: toward an improved understanding of the role of vegetated coastal habitats in sequestering CO2. Frontiers in Ecology and the Environment.

[ref-81] Medinger R, Nolte V, Pandey RV, Jost S, Ottenwälder B, Schlötterer C, Boenigk J (2010). Diversity in a hidden world: potential and limitation of next-generation sequencing for surveys of molecular diversity of eukaryotic microorganisms. Molecular Ecology.

[ref-82] Medlin L, Elwood HJ, Stickel S, Sogin ML (1988). The characterization of enzymatically amplified eukaryotic 16S-like rRNA-coding regions. Gene.

[ref-83] Menegotto A, Dambros CS, Netto SA (2019). The scale-dependent effect of environmental filters on species turnover and nestedness in an estuarine benthic Community. Ecology.

[ref-84] Meyer JL (1990). A blackwater perspective on riverine ecosystems. BioScience.

[ref-85] Millward RN, Grant A (1995). Assessing the impact of copper on nematode communities from a chronically metal-enriched estuary using pollution-induced community tolerance. Marine Pollution Bulletin.

[ref-86] Montserrat JM, Rosa CE, Sandrini JZ, Marins LF, Bianchini A, Geracitano (2003). Annelids and nematodes as sentinels of environmental pollution. Comments on Toxicology.

[ref-87] Mulik J, Sukumaran S, Srinivas T (2020). Factors structuring spatio-temporal dynamics of macrobenthic communities of three differently modified tropical estuaries. Marine Pollution Bulletin.

[ref-88] Naro-Maciel E, Ingala MR, Werner IE, Reid BN, Fitzgerald AM (2022). COI amplicon sequence data of environmental DNA collected from the Bronx River Estuary, New York City. Metabarcoding and Metagenomics.

[ref-89] Nascimento FJA, Karlson AML, Elmgren R (2008). Settling blooms of filamentous cyanobacteria as food for meiofauna communities. Limnology and Oceanography.

[ref-90] Nascimento FJA, Lallias D, Bik HM, Creer S (2018). Sample size effects on the assessment of eukaryotic diversity and community structure in aquatic sediments using high-throughput sequencing. Scientific Reports.

[ref-91] NAS National Academy of Sciences (1977). Copper. Committee on medical and biologic effects of environmental pollutants, National Research Council.

[ref-92] Neto JM, Bernardino AF, Netto SA (2021). Rhodolith density influences sedimentar organic matter quality and biochemical composition, and nematode diversity. Marine Environmental Research.

[ref-93] Oksanen J, Simpson GL, Blanchet FG, Kindt R, Legendre P, Minchin PR, O’Hara RB, Solymos P, Stevens MHH, Szoecs E, Wagner H, Barbour M, Bedward M, Bolker B, Borcard D, Carvalho G, Chirico M, Caceres MD, Durand S, Evangelista HBA, John RF, Friendly M, Furneaux B, Hannigan G, Hill MO, Lahti L, McGlinn D, Ouellette M-H, Cunha ER, Smith T, Stier A, Braak CJFT, Weedon J (2022). https://CRAN.R-project.org/package=vegan.

[ref-94] Olsgard F (1999). Effects of copper conta, mination on recolonisation of subtidal marine soft sediments—an experimental field study. Marine Pollution Bulletin.

[ref-95] Paarsons TR, Takahashi M, Hargrave B (1984). Biological océanographie processes.

[ref-96] Pawlowski J, Bruce K, Panksep K, Aguirre FI, Amalfitano S, Apothéloz-Perret-Gentil L, Baussant T, Bouchez A, Carugati L, Cermakova K, Cordier T, Corinaldesi C, Costa FO, Danovaro R, Dell’Anno A, Duarte S, Eisendle U, Ferrari BJD, Frontalini F, Frühe L, Haegerbaeumer A, Kisand V, Krolicka A, Lanzén A, Leese F, Lejzerowicz F, Lyautey E, Maček I, Sagova-Marečková M, Pearman JK, Pochon X, Stoeck T, Vivien R, Weigand A, Fazi S (2022). Environmental DNA metabarcoding for benthic monitoring: a review of sediment sampling and DNA extraction methods. Science of The Total Environment.

[ref-97] Pedregosa F, Varoquaux G, Gramfort A, Michel V, Thirion B, Grisel O, Blondel M, Prettenhofer P, Weiss R, Dubourg V, Vanderplas J, Passos A, Cournapeau D, Brucher M, Perrot M, Duchesnay E (2011). Scikit-learn: machine learning in Python. Journal of Machine Learning Research.

[ref-98] Pendleton L, Donato DC, Murray BC, Crooks S, Jenkins WA, Sifleet S, Craft C, Fourqurean JW, Kauffman JB, Marbá N, Megonigal P, Pidgeon E, Herr D, Gordon D, Baldera A (2012). Estimating global blue carbon emissions from conversion and degradation of vegetated coastal ecosystems. PLOS ONE.

[ref-99] Quast C, Pruesse E, Yilmaz P, Gerken J, Schweer T, Yarza P, Peplies J, Glöckner FO (2013). The SILVA ribosomal RNA gene database project: improved data processing and web-based tools. Nucleic Acids Research.

[ref-100] Queiroz HM, Nóbrega GN, Ferreira TO, Almeida LS, Romero TB, Santaella ST, Bernardino AF, Otero XL (2018). The Samarco mine tailing disaster: a possible time-bomb for heavy metals contamination?. Science of the Total Environment.

[ref-101] Queiroz HM, Ying SC, Bernardino AF, Barcellos D, Nóbrega GN, Otero XL, Ferreira TO (2021). Role of Fe dynamic in release of metals at Rio Doce estuary: unfolding of a mining disaster. Marine Pollution Bulletin.

[ref-102] R Core Team (2022). https://www.R-project.org/.

[ref-103] Ruppert KM, Kline RJ, Rahman MS (2019). Past, present, and future perspectives of environmental DNA (eDNA) metabarcoding: a systematic review in methods, monitoring, and applications of global eDNA. Global Ecology and Conservation.

[ref-104] Rutledge PA, Fleeger JW (1993). Abundance and seasonality of meiofauna, including harpacticoid copepod species, associated with stems of the salt-marsh cord grass. Estuaries.

[ref-105] Steyaert M, Priestley V, Osborne O, Herraiz A, Arnold R, Savolainen V (2020). Advances in metabarcoding techniques bring us closer to reliable monitoring of the marine benthos. Journal of Applied Ecology.

[ref-106] Stoeck T, Bass D, Nebel M, Christen R, Jones MDM, Breiner H-W, Richards TA (2010). Multiple marker parallel tag environmental DNA sequencing reveals a highly complex eukaryotic community in marine anoxic water. Molecular Ecology.

[ref-107] Student (1908). The probable error of a mean. Biometrika.

[ref-108] Suguio K (1973). Introducão a sedimentologia.

[ref-109] Taberlet P, Coissac E, Hajibabaei M, Rieseberg LH (2012). Environmental DNA. Molecular Ecology.

[ref-110] Tang L, Li HX, Yan Y (2012). Temporal and spatial distribution of the meiobenthic community in Daya Bay, South China Sea. Ocean Science.

[ref-111] Tietjen JH (1969). The ecology of shallow water meiofauna in two new England estuaries. Oecologia.

[ref-112] Trannum HC, Olsgard F, Skei JM, Indrehus J, Øveras S, Eriksen J (2004). Effects of copper, cadmium and contaminated harbour sediments on recolonisation of soft-bottom communities. Journal of Experimental Marine Biology and Ecology.

[ref-113] Tukey JW (1949). Comparing individual means in the analysis of variance. Biometrics.

[ref-114] Underwood AJ (1997). Experiments in ecology. Their logical design and interpretation using analysis of variance.

[ref-115] USEPA United States Environmental Protection Agency (1996). Method 3052. Microwave assisted acid digestion of siliceous and organically based matrices.

[ref-116] Valentini A, Pompanom F, Taberlet P (2009). DNA barcoding for ecologists. Trends in Ecology and Evolution.

[ref-117] Varzim CS, Hadlich HL, Andrades R, Mazzuco ACA, Bernardino AF (2019). Tracing pollution in estuarine benthic organisms and its impacts on food webs of the Vitoria Bay estuary. Estuarine, Coastal and Shelf Science.

[ref-118] Wakkaf T, Allouche M, Arrath AH, Mansour L, Alwasel S, Ansari KGMT, Beyrem H, Sellami B, Boufahja F (2020). The individual and combined effects of cadmium, polyvinyl chloride (PVC) microplastics and their polyalkylamines modified forms on meiobenthic features in a microcosm. Environmental Pollution.

[ref-119] Walters K, Bell SS (1994). Significance of copepod emergence to benthic, pelagic, and phytal linkages in a subtidal seagrass bed. Marine Ecology Progress Series.

[ref-120] Ward TJ, Jacoby CA (1992). A strategy for assessment and management of marine ecosystems: baseline and monitoring studies in Jervis Bay, a temperate Australian embayment. Marine Polluution Bulletin.

[ref-121] Won EJ, Lee Y, Gang Y, Kim M-S, Kim CJ, Kim H-E, Shin K-H (2018). Chronic adverse effects of oil dispersed sediments on growth, hatching, and reproduction of benthic copepods: indirect exposure for long-term tests. Marine Environmental Research.

