# Supplementary material for: Transition of an estuarine benthic meiofauna assemblage 1.7 and 2.8 years after a mining disaster
Source: PeerJ. 2023 Mar 14;11:e14992. doi: 10.7717/peerj.14992 (PMC10022502; doi:10.7717/peerj.14992)
Supplement: Supplemental Information 3 — Results of Student’s t-test comparing environmental variables between 2017 and 2018 sampling in the Rio Doce estuary. [file peerj-11-14992-s003.docx]

| **Variable** | **df** | **t** | **p** |
| --- | --- | --- | --- |
| TOM | 21 | 0.629 | 0.646 |
| %Sand | 21 | -0.984 | 0.532 |
| Al | 21 | 3.396 | **<0.001** |
| As | 21 | 0.689 | 0.536 |
| Ba | 21 | 2.956 | **<0.001** |
| Cd | 21 | 3.271 | **<0.001** |
| Co | 21 | 1.989 | **0.004** |
| Cr | 21 | 3.709 | **<0.001** |
| Cu | 21 | 3.443 | **<0.001** |
| Fe | 21 | 4.243 | **<0.001** |
| Mn | 21 | 2.327 | **<0.001** |
| Ni | 21 | 2.347 | **<0.001** |
| Pb | 21 | 5.870 | **<0.001** |
| Zn | 21 | 1.719 | **0.009** |
| Phylogenetic Diversity | 21 | 15.609 | **<0.001** |
| Number of Sequences | 21 | 11.147 | **<0.001** |
