# Supplementary material for: Transition of an estuarine benthic meiofauna assemblage 1.7 and 2.8 years after a mining disaster
Source: PeerJ. 2023 Mar 14;11:e14992. doi: 10.7717/peerj.14992 (PMC10022502; doi:10.7717/peerj.14992)
Supplement: Supplemental Information 4 — Latitude, Longitude, salinity, and number of sequences (after filtering) identified as meiofaunal metazoans in each sampled station in the 2018 assessment in the Rio Doce estuary. [file peerj-11-14992-s004.docx]

| **Sample Name** | **Lat** | **Long** | **Salinity(ppt)** | **Number of Sequences after filtering** |
| --- | --- | --- | --- | --- |
| ST4 | 19°38'38.80"S | 39°49'16.40"W | 0.1 | 7,196 |
| ST6 | 19°38'27.10"S | 39°48'44.10"W | 0.1 | 5,890 |
| ST8 | 19°38'16.30"S | 39°49'4.80"W | 0.1 | 13,098 |
| ST9 | 19°38'16.40"S | 39°48'41.80"W | 0.1 | 16,508 |
| ST11 | 19°38'20.60"S | 39°49'2.00"W | 0.7 | 14,764 |
| ST13 | 19°38'16.20"S | 39°48'52.30"W | 0.1 | 4,758 |
| ST14 | 19°37'51.00"S | 39°49'0.50"W | 0.1 | 23,250 |
| ST15 | 19°38'49.40"S | 39°48'55.59"W | 0.1 | 5,947 |
| ST18 | 19°37'28.30"S | 39°49'11.10"W | 0.1 | 21,454 |
| ST19 | 19°38'30.70"S | 39°49'25.70"W | 0.1 | 7,762 |
