# Supplementary material for: Transition of an estuarine benthic meiofauna assemblage 1.7 and 2.8 years after a mining disaster
Source: PeerJ. 2023 Mar 14;11:e14992. doi: 10.7717/peerj.14992 (PMC10022502; doi:10.7717/peerj.14992)
Supplement: Supplemental Information 5 — Results of ANOVA comparing the frequencies of meiofaunal representative sequences obtained in the 2018 assessment in the Rio Doce estuary. [file peerj-11-14992-s005.docx]

| **Source of Variation** | **df** | **F** | **p** |
| --- | --- | --- | --- |
| Between Groups | 9 | 12.715 | **<0.001** |
| Residual | 90 |  |  |
| Total | 99 |  |  |
